# The *AaDREB1* Transcription Factor from the Cold-Tolerant Plant *Adonis amurensis* Enhances Abiotic Stress Tolerance in Transgenic Plant

**DOI:** 10.3390/ijms17040611

**Published:** 2016-04-22

**Authors:** Jun-Mei Zong, Xiao-Wei Li, Yuan-Hang Zhou, Fa-Wei Wang, Nan Wang, Yuan-Yuan Dong, Yan-Xi Yuan, Huan Chen, Xiu-Ming Liu, Na Yao, Hai-Yan Li

**Affiliations:** College of Life Sciences, Engineering Research Center of the Chinese Ministry of Education for Bioreactor and Pharmaceutical Development, Jilin Agricultural University, Changchun 130118, Jilin, China; zjm@frontier-ag.com (J.-M.Z.); xiaoweili1206@163.com (X.-W.L.); meyuanhang@163.com (Y.-H.Z.); fw-1980@163.com (F.-W.W.); wangnanlunwen@126.com (N.W.); yydong@aliyun.com (Y.-Y.D.); yuanyanxi123@163.com (Y.-X.Y.); chjlau@163.com (H.C.); xiuming1211@163.com (X.-M.L.); yaona801103@aliyun.com (N.Y.)

**Keywords:** *AaDREB1*, abiotic stress tolerance, *Adonis amurensis*, transgenic plant

## Abstract

Dehydration-responsive element binding (DREB) transcription factors (TFs) play important roles in the regulation of plant resistance to environmental stresses and can specifically bind to dehydration-responsive element/C-repeat element (DRE/CRT) proteins (G/ACCGAC) and activate expression of many stress-inducible genes. Here, we cloned and characterized a novel gene (*AaDREB1*) encoding the DREB1 transcription factor from the cold-tolerant plant *Adonis amurensis*. Quantitative real-time (qRT)-PCR results indicated that *AaDREB1* expression was induced by salt, drought, cold stress, and abscisic acid application. A yeast one-hybrid assay demonstrated that *AaDREB1* encodes a transcription activator and specifically binds to DRE/CRT. Furthermore, transgenic *Arabidopsis* and rice harboring *AaDREB1* showed enhanced tolerance to salt, drought, and low temperature. These results indicated that AaDREB1 might be useful in genetic engineering to improve plant stress tolerance.

## 1. Introduction

Plant growth and agricultural production are greatly constrained by environmental stresses, such as salinity, drought, extreme temperatures, UV irradiation and pathogen attacks. Plants, as sessile organisms, have evolved appropriate regulatory mechanisms that act at the cellular, molecular, physiological and biochemical levels to sense and rapidly adapt to stress conditions. Molecular and cellular responses to abiotic stresses involve signal perception, transduction of the signal to the cytoplasm and nucleus, alteration of gene expression and, finally, metabolic changes that lead to stress tolerance [1]. Among the stress-related genes, transcription factors (TFs) play an important role in regulating a plant’s response to stress conditions. TFs act as master switches and trigger simultaneous expression of a large number of stress-response genes that contribute to the stress-tolerance phenotype.

All dehydration-responsive element binding (DREB) proteins belong to the DREB TFs subfamily of the APETALA2/ethylene-responsive element binding proteins (AP2/EREBP) family, contain a conserved DNA-binding domain, specifically bind the promoter regions of downstream genes, activate or suppress the transcription of these genes and finally enhance plant stress tolerance [2,3,4]. DREB homologous genes have been isolated from a variety of species [5], such as *Arabidopsis* [6], *Zea mays* [7], *Gossypium hirsutum* [8], rice [9], tomato [10], hot pepper [11], soybean [12], perennial ryegrass [13] and dwarf apple [14]. Expression of dehydration-responsive element binding1/C-repeat binding factor (*DREB1*/*CBF*) genes is strongly induced by cold, but not by dehydration and high-salt stresses, whereas expression of *DREB2* genes is induced by dehydration and high-salt stresses but not by cold. These results suggest that two independent families of DRE-binding proteins function as *trans*-acting factors in two separate signal-transduction pathways under low-temperature and dehydration conditions, respectively [15,16,17,18]. Expression of CBF homologs can be induced by drought, high-salt and abscisic acid (ABA) treatment [19,20]. Overexpression of *DREB1A/CBF3* under the control of the CaMV35S promoter also increased the tolerance to drought, high-salt, and freezing stresses [15,21]. Transgenic plants overexpressing either *DREB1* or *DREB2A* genes enhanced tolerance to abiotic stress but also a marked decrease in plant height and delayed flowering time compared with wild-type plants [4,22,23,24,25].

*Adonis amurensis* is a cold-tolerant plant capable of sprouting and flowering prior to melting of ice and snow. The cold-responsive genes in this species therefore are of great research interest. Characterization of *DREB* genes from *A. amurensis* would contribute to our understanding of the molecular mechanisms of stress resistance to improve tolerance to adverse environments in economically important plants using transgenic technology. In this study, we cloned and characterized a novel gene (*AaDREB1)* from *A. amurensis*. Its expression pattern and transcription activation activity were investigated and overexpression of *AaDREB1* in *Arabidopsis thaliana* and rice imparted enhanced tolerance to drought, salinity, and low temperature.

## 2. Results

### 2.1. Isolation and Sequence Analysis of AaDREB1

**Figure 1:**
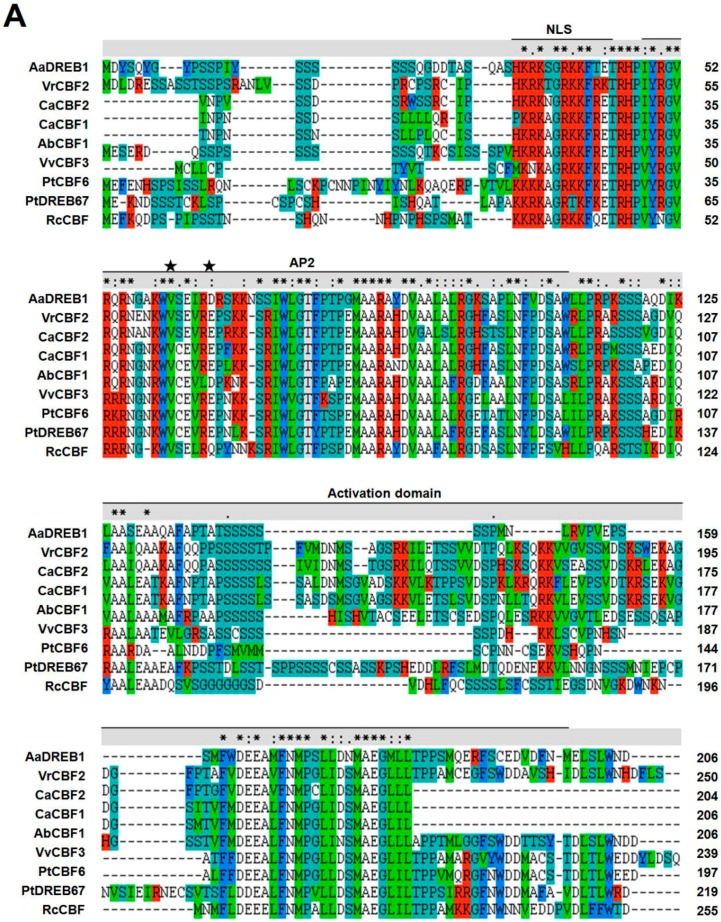

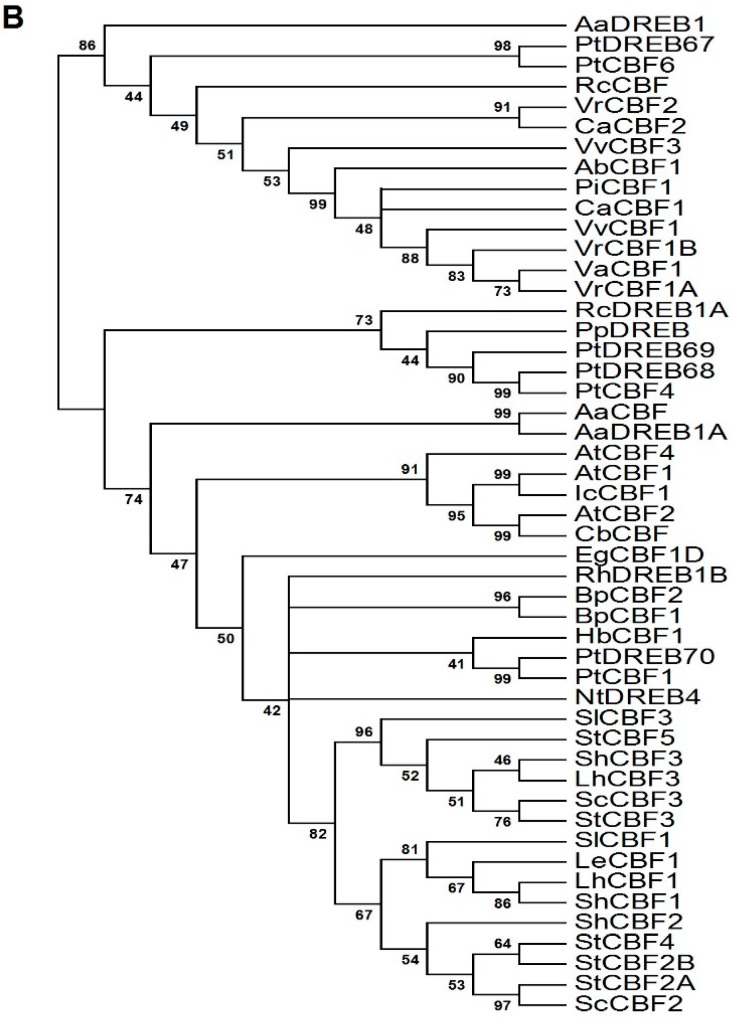
Comparison of the deduced amino acid sequence of *Adonis amurensis* dehydration-responsive element binding protein 1 (AaDREB1) and its homologs. Information of the DREB proteins of plants used here are in Supplementary Materials 1. (**A**) The sequences were aligned using CLUSTAL X. Gaps were introduced to optimize the alignment. The asterisks “*” indicate positions with a single, fully conserved residue; ‘‘:’’ indicates highly conserved positions; and “.” indicates more weakly conserved positions. Asterisks mark 14th and 19th amino acids of APETALA2/ethylene-responsive element-binding factor (AP2/ERF) domain. Dashes indicate gaps in the amino acid sequences. The column shows the score of the conservation positions. Lines above the alignment indicate the nuclear localization signal (NLS), the conserved AP2 domain, and the activation domain; identical and similar amino acids sequences are highlighted by red, deongaree, Cambridge blue and green shading respectively; (**B**) Phylogenetic analysis of *Adonis amurensis* and other plants’ DREB family proteins. Analysis, based on minimum evolution, was performed with full-length protein sequences using the AP2 transcription factor as an outgroup. DREB proteins were initially aligned using Clustal W and were used for phylogenetic analysis using MEGA version 4.1 software. The phylogenetic tree was constructed using the neighbor-joining method with 1000 bootstrap replications. Bootstrap percentages are shown at dendrogram branch points.

Sequence analysis confirmed isolation of a full-length cDNA of *AaDREB1* (GenBank accession number: ADY68770.1) encoding a protein of 206 amino acids. The amino acids from 53 to 63 comprised a putative nuclear localization signal domain; from 68 to 131 they comprised a typical AP2 domain; and the amino acids between 136 and 273 contained a possible activation domain (Figure 1A). The deduced protein encoded by *AaDREB1* also contained two conserved functional amino acids (valine and glutamic acid), located at the 14th and 19th residues in the domain and were thought to be crucial sites responsible for binding between DREB transcription factors and dehydration-responsive element (DRE) core sequences (TACCGACAT) [15] (Figure 1A). These features suggest that *AaDREB1* encodes a possible AP2/EREBP transcription factor (TF). A BLAST analysis using NCBI databases also showed that *AaDREB1* was homologous to DREB or DREB-like TFs from other plants. Cluster analysis of the deduced protein encoded by *AaDREB1* showed that the AP2 domain of this protein had high homology with PtCBF6 (ABO48367; 48.84%), PtDREB67 (XP–002328068; 47.27%), and RcCBF (XP–002532187; 43.75%) (Figure 1B).

### 2.2. Expression Patterns of AaDREB1

**Figure 2:**
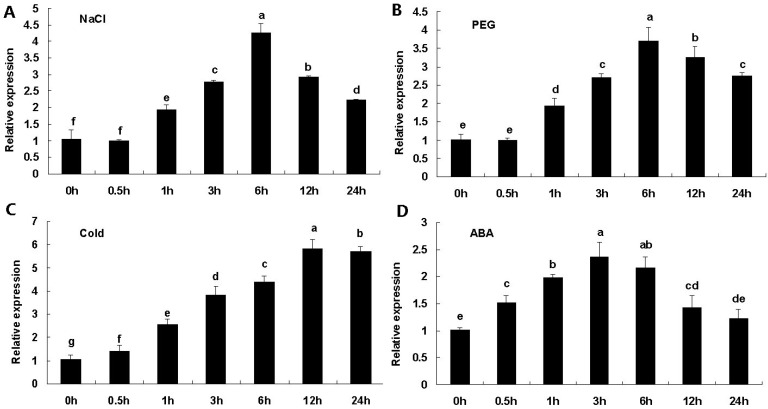
*AaDREB1* expression patterns in response to different stress treatments. Leaves were collected at 0, 0.5, 1, 3, 6, 12 and 24 h after the initiation of high salinity (**A**), drought (**B**), low temperature (**C**) and abscisic acid (**D**). *β-Actin* gene was used as an internal standard. Values are means ± standard errors of three biological repeats. Different letters on the column represent significant difference (*p* < 0.05) based on Duncan’s multiple range test.

Expression patterns of *AaDREB1* in response to the different stress treatments were analyzed (Figure 2). Under salt stress, expression of *AaDREB1* began to increase after 1 h, reached its maximum at 6 h, and then gradually decreased after 12 h (Figure 2A). Similarly, expression was also induced by drought stress, mRNA accumulation peaked at 6 h and started to decline after 12 h (Figure 2B). Under low-temperature stress, transcription of *AaDREB1* was strongly increased after 0.5 h and reached its maximum at 12 h (Figure 2C). In response to ABA treatment, *AaDREB1* mRNA was weakly induced after 0.5 h, peaked at 3 h, and started to decline after 6 h (Figure 2D). Thus, *AaDREB1* was induced by salt, drought, cold stresses, and ABA application.

### 2.3. Analysis of Trans-Activation Activity of AaDREB1

**Figure 3:**
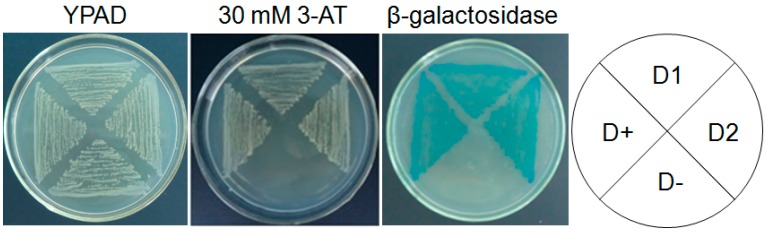
Yeast one-hybrid assays of AaDREB1. All yeast transformants carrying dual reporter genes were examined for growth in the presence of 3-AT, and β-galactosidase activity. The pAD-*AaDREB1* was transformed into DRE yeast (D1, D2), with the DRE yeast containing the pAD-*AtCBF3* as the positive control (D+) and the mDRE yeast containing the pAD-*AtCBF3* as the negative control (D-). Transformants were streaked on YPAD medium or SD/-Try with 30 mM 3-AT and grown at 30 °C for 2 days to observe growth and β-galactosidase activities. **Left** panel, YPAD medium; **middle** panel, SD/-Try medium containing 30 mM 3-AT for growth analysis; **right** panel, β-galactosidase activity analysis results.

All transformants grew normally on selective medium (SD/-Try) medium. The β-galactosidase activity assay showed that the pAD-*AaDREB1* transformants of the dehydration-responsive element (DRE) yeast turned blue, while the mutant DRE (mDRE) transformants did not (Figure 3). Thus, heterogeneous expression of *AaDREB1* promoted expression of the *lacZ* gene in wild-type DRE yeast but not in mutant DRE yeast, indicating that *AaDREB1* encodes a transcription factor that can specifically bind to the DRE sequence in the promoter region and activate transcription of the downstream genes *in vivo*.

### 2.4. Overexpression of AaDREB1 Results in Enhanced Salt, Drought, and Cold Tolerance in Arabidopsis

**Figure 4:**
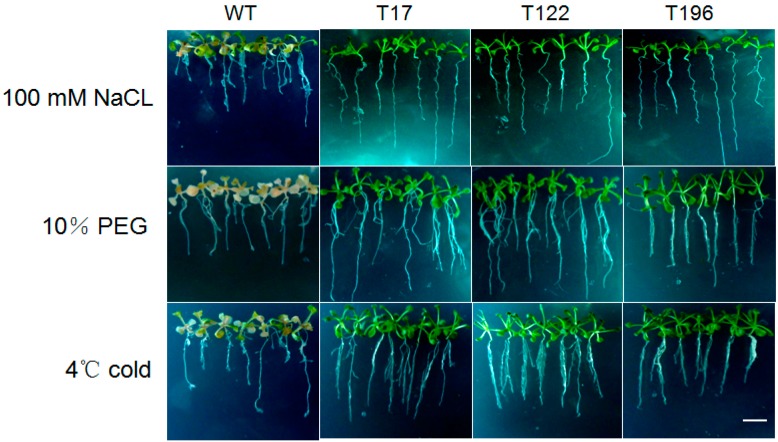
Stress-tolerance assays of *AaDREB1* overexpressing transgenic *Arabidopsis*. Ten-day-old seedlings of *AaDREB1* transgenic lines (T17, T122, T196) and the empty vector control plants (WT) were treated with either 100 mM NaCl for 12 days, 10% PEG for 10 days, or exposed to 4 °C for 20 h, then grown under normal growth conditions for 3 days. Scale bar represents 1.5 cm.

To examine the function of *AaDREB1* in plant stress responses, the salt, drought, and cold tolerances of *AaDREB1*-overexpressing transgenic *Arabidopsis* lines and control plants were assessed. The wild-type plants withered and leaves were whitened, whereas the transgenic lines grew well with green leaves. Of the seedlings exposed to −4 °C for 20 h, the controls withered and died (Figure 4). These results indicated that overexpression of *AaDREB1* in *Arabidopsis* greatly enhanced plant tolerance to salt, drought, and cold stresses, and that *AaDREB1* might play important roles in plant stress signal transduction.

### 2.5. Overexpression of AaDREB1 Improved Salt Tolerance of Transgenic Rice Plants

**Figure 5:**
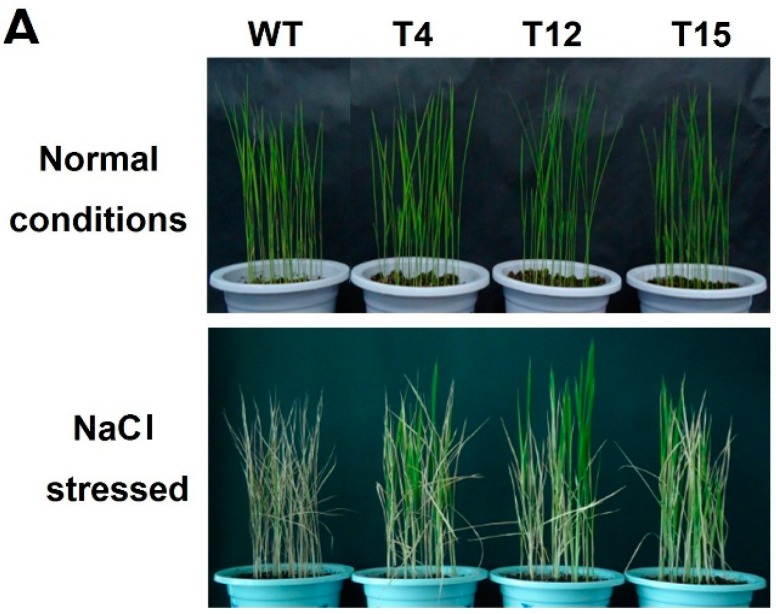

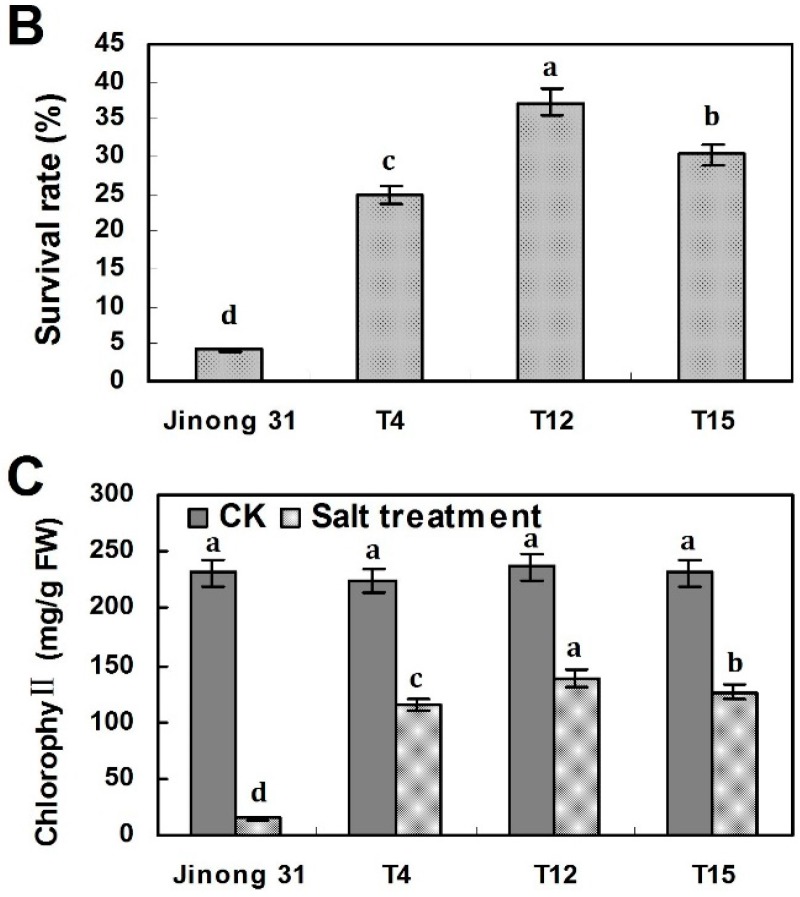
Salt-tolerance analyses of wild-type plants and *AaDREB1* transgenic rice. (**A**) The phenotype of two-week-old *AaDREB1* transgenic lines and wide-type rice plants grown in the glasshouse under normal growth conditions before being transferred to 150 mM NaCl for 16 days; (**B**) Survival rates of transgenic and wild-type plants after high-salt treatment; (**C**) The chlorophyll content of the leaves of transgenic and wild-type plants after high-salt treatment and under normal growth conditions. FW, fresh weight. Values are means ± standard errors of three biological repeats. Different letters on the column represent significant difference (*p* < 0.05) based on Duncan’s multiple range test.

The phenotypes and chlorophyll contents of *AaDREB1*-overexpressing transgenic rice were similar to those of wild-type rice plants grown under unstressed conditions (220–240 mg·g^−1^ FW) (Figure 5A,C). When plants were treated with 150 mM NaCl, most wild-type plants withered and died (4.2% survival rate, 4/94), whereas 25% (23/92), 37.3% (34/91), and 30.2% (29/96) of transgenic lines T4, T12, and T15 remained green and survived (Figure 5A,B). The average chlorophyll contents of transgenic lines (115–140 mg·g^−1^ FW) were significantly higher than that of the wild-type (15 mg·g^−1^ FW) (Figure 5C) in response to high salt treatment. These results indicated that overexpression of *AaDREB1* enhanced salt tolerance of transgenic rice during the seedling stage.

### 2.6. Overexpression of AaDREB1 Improved Drought Tolerance of Transgenic Rice Plants

**Figure 6:**
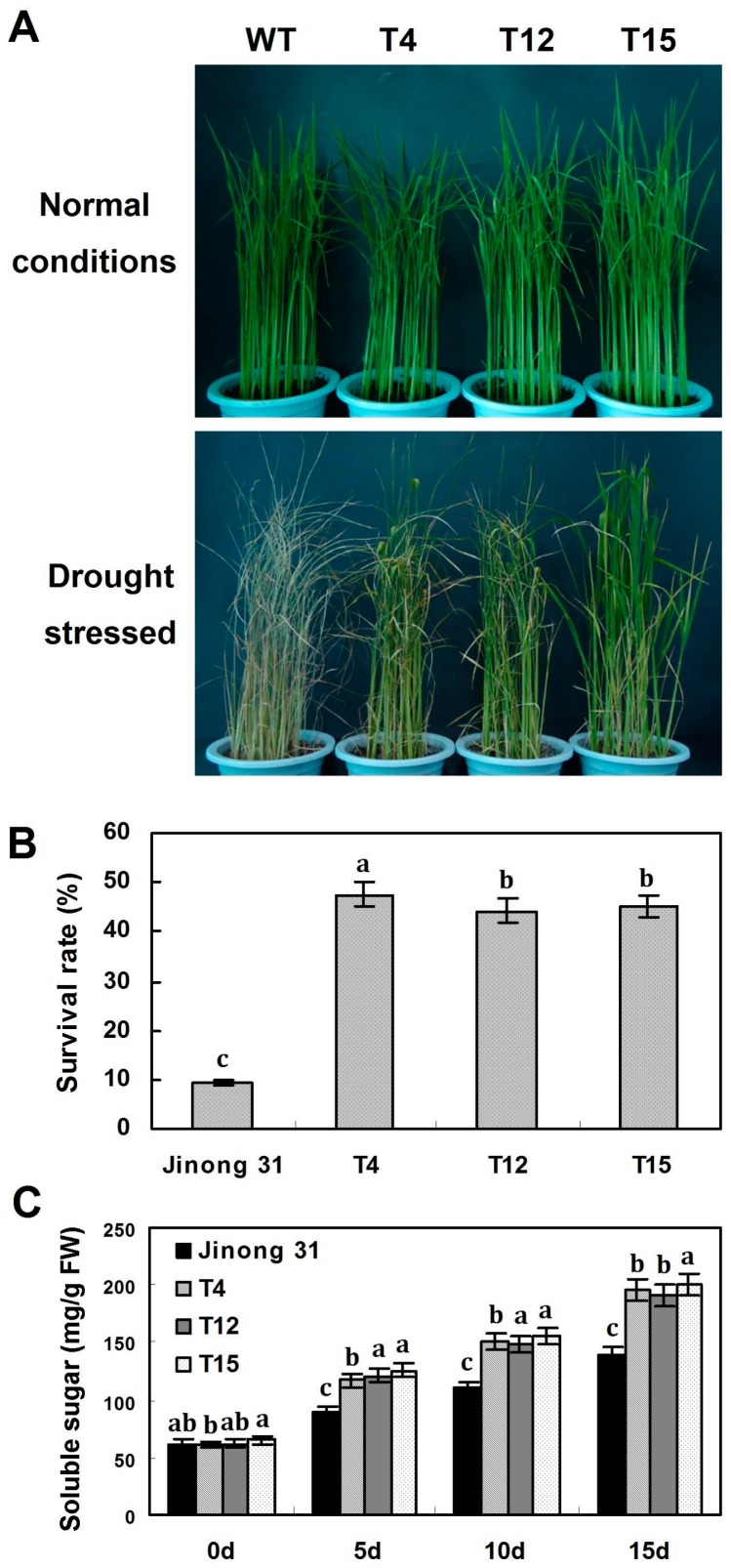
Drought tolerance analyses of wild-type plants and *AaDREB1* transgenic rice. (**A**) The phenotype of transgenic and wild-type rice seedlings growing in soil in the glasshouse under normal growth conditions for seven weeks then were deprived of water for 15 days at 25 °C; (**B**) Survival rate of *AaDREB1* transgenic and wild-type plants after drought treatment; (**C**) Soluble sugar content of transgenic and wild-type plants. FW, fresh weight. Values are means ± standard errors of three biological repeats. Different letters on the column represent significant difference (*p* < 0.05) based on Duncan’s multiple range test.

As shown in Figure 6A, seven-week-old T_1_ transgenic plants deprived of water for 12 h exhibited increased tolerance to drought stress, and the transgenic lines survived with parts of the leaves coiled, whereas the wild-type plants withered with stems bent and leaves coiled severely. Survival rates were 47.6% (39/82) for *35S::AaDREB1* transgenic lines T4, and 44.3% (43/97) and 45.1% (41/91) for *35S::AaDREB1* transgenic lines T12 and T15, respectively, compared with 9.2% (8/87) for wild-type plants (Figure 6B). To evaluate physiological changes in seven-week-old transgenic plants deprived of water for 15 days, soluble sugar contents in the leaves of different transgenic and wild-type plants were compared. Under control conditions, the soluble sugar contents in leaves were similar for different transgenic and wild-type plants (about 62 mg·g^−1^ FW). However, following drought treatment, the soluble sugar levels in transgenic plants increased after 5 days, and reached 191–201 mg·g^−1^ FW at 15 days, whereas those in wild-type plants at the same developmental stage reached only 139 mg·g^−1^ FW at 15 days. Statistical analysis showed that soluble sugar contents of transgenic rice plants were significantly higher than those of the wild-type at 5, 10, 15 and 20 days (Figure 6C).

### 2.7. Overexpression of AaDREB1 Enhanced Freezing Tolerance of Transgenic Rice Plants

**Figure 7:**
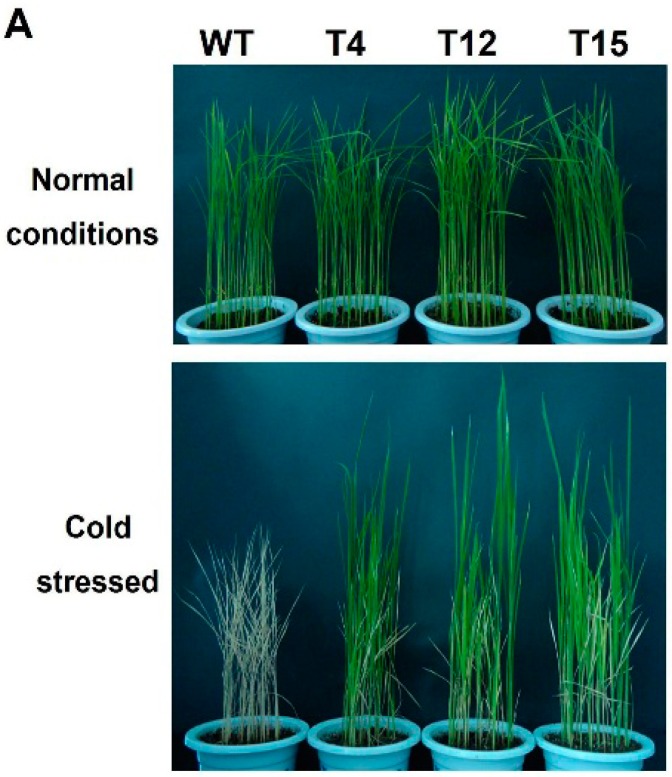

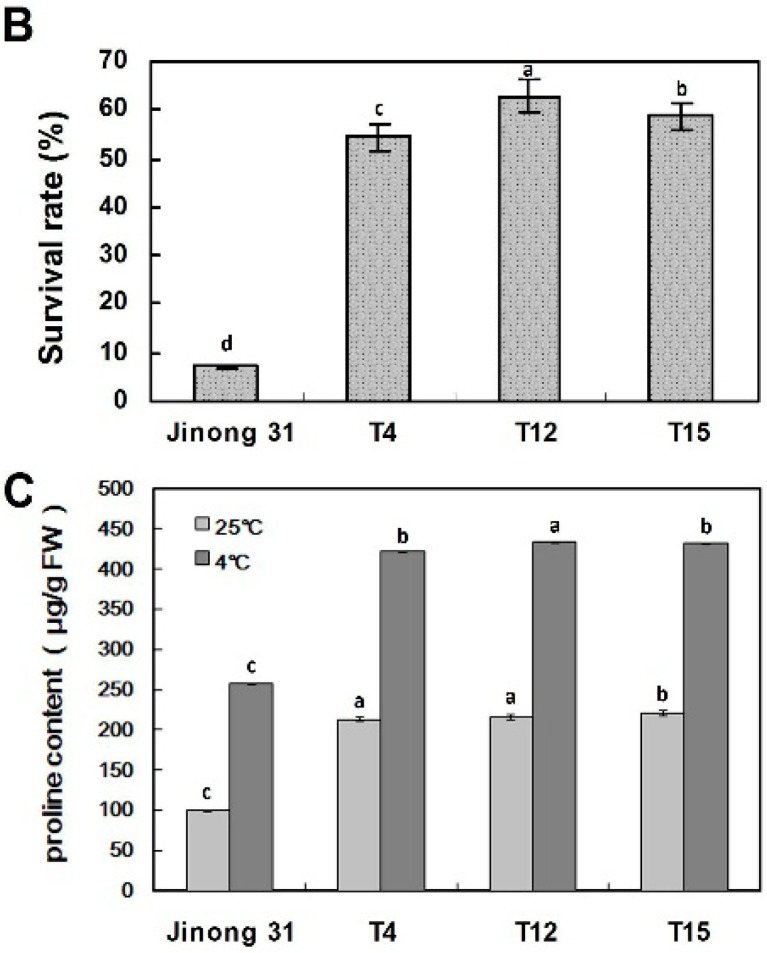
Low-temperature tolerance analyses of *AaDREB1* transgenic and wild-type plants. (**A**) The phenotype of three-week-old *AaDREB1* transgenic lines and wide-type rice plants grown in the glasshouse under normal growth conditions before being exposed to 4 °C for 12 days followed by recovery at 25 °C for 5 days; (**B**) Survival rate of the control and three transgenic lines after cold treatment; (**C**) Proline concentration in three-week-old transgenic and wild-type rice plants after cold treatment. FW, fresh weight. Values are means ± standard errors of three biological repeats. Different letters on the column represent significant difference (*p* < 0.05) based on Duncan’s multiple range test.

T_1_ transgenic and wild-type plants were treated and allowed to recover prior to assessment of survival rates and free proline content analysis. After treatment with 4 °C for 12 days, wild-type plants showed retarded growth, whereas all transgenic lines grew well and only the leaf tips coiled slightly (Figure 7A). The survival rates of the transgenic lines were 54.5%, (54/99) for T4, 62.9% (61/97) for T12 and 58.7% (54/92) for T15, respectively, which were significantly higher than that of the wild-type plants (7.3%, 7/96; *p* ≤ 0.05) (Figure 7B). In addition, under normal growth conditions, free proline concentrations in the transgenic plants were approximately two-fold higher than the control plants (Figure 7C). This indicated that overexpression of *AaDREB1* in rice improved freezing stress tolerance.

## 3. Discussion

In this study, we isolated and characterized an *AaDREB1* gene from the cold-tolerant plant *A. amurensis*. The deduced amino acid sequence of *AaDREB1* contained a nuclear localization signal (NLS), an AP2 DNA-binding domain, and an activation domain flanking this domain, which was conserved in all of the other DREB1 transcription factors [2]. Sequence Blast analysis and classification revealed that AaDREB1 belongs to the DREB subfamily of the AP2/EREBP TF family and was placed in the A-1 group [26], which specifically binds to DRE/CRT elements and plays important roles in plant ABA-independent abiotic stress signaling pathways. The *in vivo* binding assay in yeast showed that AaDREB1 could specifically bind the DRE motif in the promoter and activate transcription of the fused reporter genes. Thus, AaDREB1 is a DRE-binding transcription factor in *A. amurensis*.

Expression of *AaDREB1* in *A. amurensis* is induced by high salinity, drought, cold and ABA application. This pattern of expression is similar to that of *OsDREB1F* in rice [27], CBF1 in *Arabidopsis* [28], and *ZmDREB2A* in maize [7], but differs from that of *OsDREB1A/B* in rice [29], *ZmDREB1A* in maize [30], *AtDREB1A/B/C* in *Arabidopsis* [6,15] and *GhDREB1* in cotton [31]. AaDREB1 responded to exogenous ABA, implying that it functions in an ABA-dependent pathway. The different expression patterns suggest that AaDREB1 may function differently from its homologs. The ABA-inducible characteristics suggested that AaDREB1 may also participate in ABA-dependent signal transduction pathways, which could be confirmed by examining the expression of downstream genes.

Overexpression of *AaDREB1* in *Arabidopsis* and rice led to greatly enhanced tolerance to drought, high-salt and low-temperature conditions without growth retardation (Figure 4, Figure 5, Figure 6 and Figure 7). These results indicated that overexpression of *AaDREB1* may play important roles in plant stress signaling transduction. Overexpression of *OsDREB1F* in rice yielded similar results [28], while Ito *et al.* [32] reported that the overexpression of *OsDREB1A*, *OsDREB1B*, *AtDREB1A*, and *AtDREB1B* in rice resulted in various levels of growth inhibition under normal conditions. However, over-expression of AaDREB1 in *Arabidopsis* and rice had no impact on growth of plants. These results suggested that *AaDREB1* probably functions differently from *OsDREB1A* and *OsDREB1B*, although all genes participated in stress signal transduction pathways.

Under drought, high-salt and low-temperature conditions, osmolytes such as various soluble sugars, in addition to free proline accumulate and play multiple roles in plant adaptation to stress [32,33]. Overexpression of *GhDREB* in transgenic wheat plants was associated with increased soluble sugar levels compared with wild-type plants under drought conditions [8], and overexpression of *GhDREB1* in transgenic tobacco plants resulted in a higher level of free proline than in wild-type plants under low temperature conditions [31]. In our study, *AaDREB1* transgenic plants maintained higher chlorophyll levels than control plants under high-salt stress (Figure 5C). A possible explanation was that overexpression of *AaDREB1* activated expression of downstream genes that prevented chlorophyll decomposition, thus maintaining normal photosynthesis, and improving tolerance to high-salt stress [34]. Moreover, the AaDREB1 transgenic plants under normal conditions contained more soluble sugars than wild-type rice plants did, and the difference reached a significant level. *AaDREB1* transgenic plants also accumulated higher levels of soluble sugars than wild-type plants under drought stress conditions (Figure 6C), suggesting that overexpression of *AaDREB1* activated the expression of downstream genes involved in sugar biosynthesis, which in turn enhanced tolerance to drought stress in transgenic plants [12,32]. Under cold stress and normal growth temperatures, the free proline concentration in *AaDREB1*-overexpressing plants was approximately two-fold higher than that of control plants (Figure 7C). This might suggest a direct regulation of proline production processes by AaDREB1, and that AaDREB1 acts as a transcriptional activator in transgenic rice, which activates certain downstream genes involved in proline synthesis, conferring higher plant tolerance to cold stress and protecting the photosynthetic apparatus of plant cells through osmotic regulation [31]. Further research should be performed to study the mechanisms by which the *AaDREB1* gene regulates plant tolerance to drought, salt and low-temperature stresses. However, the current results strongly suggest that *AaDREB1* is an ideal candidate gene for the genetic manipulation of crops with the goal of abiotic stress-tolerance breeding.

## 4. Experimental Section

### 4.1. Plant Materials and Stress Treatments

Seedlings of *A. amurensis* were grown in pots (13 cm diameter × 16 cm high) containing compost soil (leaf-mould:stable-litter:sand; 1:1:1) for 30 days, with a light/dark photoperiod of 16/8 h at 25 °C, prior to treatment. For the low-temperature treatment, uniformly developed seedlings were treated at 4 °C. For other treatments, seedlings of *A. amurensis* were irrigated with 500 mL solutions containing 200 mM NaCl, 8% polyethylene glycol (PEG) 8000, or 100 μM ABA, respectively. The leaves of the plants were harvested at 0, 0.5, 1, 3, 6, 12 and 24 h after treatment, directly put into liquid nitrogen and stored at −80 °C for qRT-PCR analysis. The experiment was repeated three times. Plant materials were sampled consistently at an appointed time after stress treatment for gene expression.

### 4.2. Isolation of AaDREB1 from A. amurensis by RT-PCR and 5’ and 3’ RACE

Bioinformatics methods and RT-PCR and 5’ and 3’ rapid-amplification of cDNA ends (RACE) procedures were used to clone the full-length cDNA of the AaDREB1 protein from *A. amurensis*. To isolate the middle sequence of *AaDREB1*, degenerate primers were designed based on the conserved AP2 domain of the following dicotyledon *DREB* genes: AtDREB1b/AtCBF1 (At4g25490), AtDREB1c/AtCBF2 (At4g25470), AtDREB1a/AtCBF3 (At4g25480), AtDREB1d/AtCBF4 (At5g51990), AtDREB1F/DDF1 (At1g12610), AtDREB1E/DDF2 (At1g63030), ScCBF3 (ACB45092.1), RcDREB1A (XP_002509702.1), PpDREB (ABR19831.1), EgCBF1D (ACF15447.1), NtDREB4 (ACE73696.1), RhDREB1B (ACI42860.1) and PtDREB69 (XP_002298067.1). The multiple alignment of these genes above is in Figure S1. The primer sequences are as follows: forward primer, 5’-TTYMRDGAGACDMGDCACCC-3’; reverse primer, 5’-ARRAGMADNCCYTCNGCCAT3’. In the sequences, Y denotes C or T, M denotes A or C, R denotes A or G, D denotes A, G or T, and N denotes any nucleotide. Total RNA was extracted from leaves of *A. amurensis* that were treated at 4 °C for 2 h using TRIzol^®^ reagent (Invitrogen, Carlsbad, CA, USA) and then treated with RNase-free DNase (Invitrogen). One microgram of treated RNA was used for first-strand cDNA synthesis and PCR. The annealing temperature in PCRs was 54 °C. The PCR products were cloned into the pGEM^®^-T Easy Vector (Promega, New York, NY, USA) and sequenced. Specific primers were designed for rapid amplification of the cDNA end according to the sequence information of the partial cDNA fragment to obtain the full-length sequence of the gene. For 5’ RACE, two antisense gene-specific primers (GSP) were designed: GSP1F (5’-ATCTTCGTCGTCGTCTTCCC-3’) and GSP2F (5’-TTCCCTCTGAGTGCCAAAGC-3’). Primers for 3’ RACE were: GSP1R (5’-TTCTGGGATGAGGAGGCAAT-3’) and GSP2R (5’-GGGAATGTGCCTAGCCATAT-3’). The RACE reactions were performed with the GeneRacer™ kit (Invitrogen). The RACE products were cloned and sequenced. A full-length cDNA sequence was obtained by combining the 5’ RACE, cDNA and 3’ RACE fragments. The full-length cDNA sequence of *AaDREB1* was amplified by PCR using the primers GSP3F (5’-ATGGATTATTCGCAGTACGG-3’) and GSP3R (5’-TTAATCGTTCCACAAGGACA-3’).

### 4.3. Quantitative Real-Time PCR Analysis

Total RNA was extracted from plants using the RNA Plant Plus Reagent (Tiangen, Changchun, China) according to the manufacturer protocol and cDNA was reverse transcribed using M-MLV reverse transcriptase (TaKaRa, Changchun, China). Quantitative real-time PCR analysis was performed using SYBR Green I dye (TaKaRa, Changchun, China) and a real-time PCR machine (Applied Biosystems 7500, Foster City, CA, USA). The qRT-PCR cycling stages consisted of initial denaturation at 95 °C (30 s), followed by 40 cycles of 95 °C (5 s), 58 °C (34 s), and a final melting curve stage of 95 °C (15 s), 60 °C (1 min), and 95 °C (15 s). The fluorescence signal was recorded during the strand elongation step at 58 °C and the melting curve stage at every 0.3 °C temperature ramp. Samples for qRT-PCR were run in three biological replicates and three technical replicates. Relative gene expression was calculated using the comparative DDCT method according to Livak and Schmittgen [35]. The *β-actin* gene was chosen as an internal control, specific primers used here were Actin-F: 5’-ACTGTGCCAATCTACGAGGG-3’ and Actin-R: 5’-TCTTACAATTTCCCGCTCTG-3’. Specific primers *AaDREB1-qF*: 5’-TATCAAACTCGCTGCTTCT-3’ and *AaDREB1-qR*: 5’-TCATCCCAGAACATAGAAG-3’ were used to amplify the *AaDREB1* gene. The experiment was repeated three times, and the results from three samples were averaged.

### 4.4. Comparison of the Deduced Amino Acid Sequence of Adonis Amurensis AaDREB1 and Its Homologs

The sequences were aligned using ClustalX. DREB proteins were initially aligned using ClustalW and were used for phylogenetic analysis using MEGA version 4.1. The phylogenetic tree was constructed using the neighbor-joining method with 1000 bootstrap replications. Information regarding the DREB proteins of plants used here are in Supplementary Materials 1.

### 4.5. In Vivo Dehydration-Responsive Element (DRE)-Binding and Transactivation Activity Using a Yeast One-Hybrid System

To analyze DRE-binding activity and transactivation activity of the isolated cDNA clone, the full-length cDNA insert was cloned into the yeast expression vector pAD-GAL4-2.1, and this plasmid was transformed into DRE and mDRE strains following [25]. The DRE yeast containing pAD-*AtCBF3* was the positive control (+), and the mDRE yeast containing pAD-*AtCBF3* was the negative control (−). The transformants were incubated on yeast extract–peptone–dextrose (YPAD) medium for 2 days to examine their growth and assay β-galactosidase activity.

### 4.6. Construction of the Plant Overexpression Vector and Transformation

The *AaDREB1* gene, amplified using the primers 5’-CGGGATCCATGGATTATTCGCAGTACGG-3’ and 5’-CGAGCTCTTAATCGTTCCACAAGGACA-3’, was digested with *BamH* I (GGATCC) and *Sac* I (GAGCTC), and the entire *AaDREB1* coding region was cloned into the *BamH* I and *Sac* I sites of the binary vector pBI121 under the control of the CaMV35S promoter in the sense orientation. The plant overexpression vector for *AaDREB1*, PBI121-*AaDREB1*, was introduced into *Agrobacterium tumefaciens* strain EHA105. *Arabidopsis thaliana* and rice were used for transformation. Transformed plants carrying PBI121-*AaDREB1* were identified by PCR. T_1_ generation plants were used in all experiments.

### 4.7. Analyses of Stress Tolerance and Physiological Changes in Transgenic Arabidopsis and Rice

Three lines (T17, T122 and T196) of transgenic T_3_ *Arabidopsis* plants were selected from among the transformants overexpressing *AaDREB1* to assess tolerance to cold, drought, or salt. Seeds of both wild-type and transgenic lines were germinated on Murashige and Skoog (MS) agar medium for 10 days. The seedlings were transferred to MS medium supplemented with either 100 mM NaCl for 12 days to assess salt tolerance, or 10% PEG for 10 days to assess drought tolerance. To evaluate cold tolerance, 14-day-old seedlings were exposed to 4 °C for 20 h and then grown under normal conditions for 3 days.

Wild-type rice (*cv*. Jinong31) and transgenic rice seeds were grown in pots (13 cm diameter × 16 cm high) at 28 °C under continuous illumination. Two-week-old seedlings were used for all treatments. For salt tolerance analyses, wild-type and transgenic seedlings were irrigated with 1000 mL at two-day intervals with 150 mM NaCl for 16 days and then with water for 15 days before measurement of survival rate and chlorophyll content. The experiment was repeated three times and 25–30 plants were analyzed in each experiment. Chlorophyll content was determined following Hardwick and Baker [36]. To further examine drought tolerance and physiological changes, wild-type and transgenic seedlings were deprived of water, and leaves were collected after 0, 5, 10, and 15 days for measurement of the soluble sugar content. After drought stress for 15 days, plants were rewatered for analysis of survival rate and soluble sugar content measured as described previously by Taji *et al.* [37]. For freezing tolerance analyses, wild-type and transgenic seedlings were treated with low temperature (4 °C) for 12 days, followed by transfer to a greenhouse for recovery. After 10 days, the survival rate and proline content were analyzed following Bates *et al.* [38].

### 4.8. Statistical Analysis

Data analysis was carried out using SPSS 13.0 (SPSS, Chicago, IL, USA). The means were tested using one-way analysis of variance (ANOVA). Duncan’s tests were used for multiple comparisons between treatments. All data were represented by an average of three replicate measurements and standard error. The significance level was *p* < 0.05.

## 5. Conclusions

In this work, we cloned and characterized a novel gene (*AaDREB1)* encoding the DREB1 transcription factor from *A. amurensis*, which is a cold-tolerant plant capable of sprouting and flowering prior to melting of ice and snow. Quantitative RT-PCR results indicated that *AaDREB1* expression was induced by salt, drought, cold stress, and abscisic acid application. A yeast one-hybrid assay demonstrated that *AaDREB1* encodes a transcription activator and specifically binds to DRE/CRT. Furthermore, *AaDREB1* transgenic rice showed improved salt, drought and cold tolerance but displayed no marked decrease in plant height compared with wild-type plants.

## Supplementary Materials

Supplementary materials can be found at http://www.mdpi.com/1422-0067/17/4/611/s1.
