# Supplementary material for: The AaDREB1 Transcription Factor from the Cold-Tolerant Plant Adonis amurensis Enhances Abiotic Stress Tolerance in Transgenic Plant
Source: Int J Mol Sci. 2016 Apr 22;17(4):611. doi: 10.3390/ijms17040611 (PMC4849061; doi:10.3390/ijms17040611)
Supplement: Supplementary file 1 [file ijms-17-00611-s001.pdf]

Supplementary Materials: The *AaDREB1* Transcription Factor from the Cold-Tolerant Plant *Adonis amurensis* Enhances Abiotic Stress Tolerance in Transgenic Plant

Jun-Mei Zong, Xiao-Wei Li, Yuan-Hang Zhou, Fa-Wei Wang, Nan Wang, Yuan-Yuan Dong, Yan-Xi Yuan, Huan Chen, Xiu-Ming Liu, Na Yao and Hai-Yan Li

Supplemental File 1. The DREB proteins of plants used for phylogenetic analysis in Figure 1.

>AaDREB1gi|324983867|gb|ADY68770.1| DREB1/CBF transcription factor [Adonis amurensis]
MDYSQYGYPSSPIYSSSSSSQGDDTASQASHKRKSGRKKFTETRHPIYRGVRQRNGAKWVSEIRDRSKKN
SSIWLGTFPTPGMAARAYDVAALALRGKSAPLNFVDSAWLLPRPKSSSAQDIKLAASEAAQAFAPTATSS
SSSSSPMNLRVPVEPSSMFWDEEAMFNMPSLLDNMAEGMLLTPPSMQERFSCEDVDFNMELSLWND

>ShCBF3 gi|171191051|gb|ACB45086.1| [Solanum habrochaites]
MFYSDPRIESSSSDSIRANHSDEEVILASNNPKKPAGRKKFRETRHPVYRGVRKRNSGKWVCEVREPNKK
TRIWLGTFPTAEMAARAHDVAAIALRGRSACLNFADSAWRLPTPASSDTKDIQKAAAEAAESFRPLKSEE
EESVVKDQSTTPDDMFFMDEEALFCMPGLLTNMAEGLMVPPPQCTEMGDLVEADDMPLWSYSI

>RcDREB1B gi|255575393|ref|XP_002528599.1| [Ricinus communis]
MEFKQDPSPIPSSTNSHQNNHPNPHSPSMATKKRKAGRKKFQETRHPVYNGVRRRNGKWVSELRQPYNNK
SRIWLGTFPSPDMAARAYDVAAFALRGDSASLNFPESVHLLPQARSTSIKDIQYAALEAADQSVSGGGGG
GSDVDHLFQCSSSSLSFCSSTIEGSDNVGKDWNKNMNMFLDEEELFNMPALLDSMAEGLILTPPAMKKGF
NWNNVEDDPVDLFFWTD

>AtCBF1 gi|18416562|ref|NP_567721.1| [Arabidopsis thaliana]
MNSFSAFSEMFGSDYEPQGGDYCPTLATSCPKKPAGRKKFRETRHPIYRGVRQRNSGKWVSEVREPNKKT
RIWLGTFQTAEMAARAHDVAALALRGRSACLNFADSAWRLRIPESTCAKDIQKAAAEAALAFQDETCDTT
TTNHGLDMEETMVEAIYTPEQSEGAFYMDEETMFGMPTLLDNMAEGMLLPPPSVQWNHNYDGEGDGDVSL
WSY

>IcCBF1 gi|71983373|gb|AAZ57434.1| [Iris lactea var. chinensis]
MNSFSAFSEMLGSDYEPQGGDYCPTLATSCPKKPAGRKKFRETRHPIYRGVRQRNSGKWVSEVREPNKKT
RIWLGTFQTAEMAARAHDVAALALRGRSACLNFADSAWRLRIPESTCAKDIQKAAAEAALAFQDETCDTT
TTNHGLDMQETMVEAIYTPEQSEGAFYMDEETMFGMPTLLDNMAEGMLLPPPSVQWNHNYDGEGDGEVPL
WSY

>ScCBF2 gi|171191061|gb|ACB45094.1| [Solanum commersonii]
MFPSYYSEPLAQLSSSSSISDNSNHYSPNNNFSDEEVINLASNNPKKPAGRKKFRETRHPVFRGIRMRNS
GKWVCEVREPNKKSRIWLGTFPTAEMAARAHDVAALALRGRSACLNFADSVWRLPIPASSNSKDIQKAAA
EAAEIFRPSSLEEESEEVSGECSNTTTPETPEKALSMNEEAQVNSFFMDEEALFYMPGLIANMAEGLMLP

LPQCLEIGDYVEADHAYISLWNYSI

>AtCBF2 gi|157273079|gb|ABV27118.1| [Arabidopsis thaliana]
MNSCSAFSEMFGSDYESPVSSGGDYSPKLATSCPKKPAGRKKFRETRHPIYRGVRQRNSGKWVCELREPN
KKTRIWLGTFQTAEMAARAHDVAAIALRGRSACLNFADSAWRLRIPESTCAKEIQKAAAEAASNFQDEMC
HMTTDAHGLDMEETLVEAIYTPEQSQDAFYMDEEAMLGMSSLLDNMAEGMLLPSPSVQWNYNFDVEGDDD
VSLWSY

>CbCBF gi|98980426|gb|AAY21899.2| [Chorispora bungeana]
MNSFSAFSEMFGSDYESPVSSGGDYSPKLATSCPKKPAGRKKFRETRHPIYRGVRQRNSGKWVCELREPN
KKTRIWLGTFQTAEMAARAHDVAAIALRDRSACLNFADSAWRLRIPESTCAKEIQKAAAEAALNFQDEMC
HMTTDAHGLDMEETLVEAIYTPEQSQDAFYMDEEAMLGMSSLLDNMAEGMLLPSPSVQWNYNFDVKGDDD
MSLWSY

>ScCBF3 gi|171191059|gb|ACB45092.1| [Solanum commersonii]
MDIFRSYYSDPQLESSSSFSDTTTYNSPNRANHSDEEVILASNNPKKPAGRKKFRETRHPVYRGVRMRNS
GKWVCEVREPNKKTRIWLGTFPTAEMAARAHDVAAIALRGRSACLNFADSAWRLPTPASSDTKDIQKAAA
EAAKSFRPLKSEEESVVTTGDQTSTPDDMFFMDEEALFCMPGLLTNMAEGLMVPPPQCTEMGDHVEADDM
PLWSYSI

>AaDREB1A gi|294335527|gb|ADE62311.1| [Ageratina adenophora]
MATLIQFNTPYTSLSADNIPTTESSSTSDYSTGTSFSDEEVMLASKNPKKRAGRKKFRETRHPVYRGVRR
RDSGKWVCEVREPNKKTRVWLGTYPTADMAARAHDVAALAMRGRSACLNFADSLWRLPIPESSNVKDIQK
AAVKAAEAFRPTETDVAVIEESNELTGNVFCVDDESIFEMQGFLADMAEGMMLPPPRTIEYDNCQDDLEF
FVDASLWSF

>AaCBF gi|125950487|gb|ABN58746.1| [Ageratina adenophora]
MATLIPFNTPYTSLSADNIPTTESSSTSDYSTGTSFSDEEVMLASKNPKKRAGRKKFRETRHPVYRGVRR
RDSGKWVCEVREPNKKTRVWLGTYPTADMAARAHDVAALAMRGRSACLNFADSLWRLPIPESSNVKDIQK
AAVKAAEAFRPTETDVAVIEESNELTGNVFCVDDESIFEMQGFLADMAEGMMLPPPRTIEYDNCQDDLEF
FVDASLWSF

>PpDREB gi|148927433|gb|ABR19831.1| [Prunus persica]
MDMFSAQLSDSPDQPESSSFSDASVTTLPASSSDENVILASSRPKKRAGRRVFKETRHPVYRGVRRRNNN
KWVCELREPNNKKSRIWLGTYPTAEMAARAHDVAALAFRGKLACINFADSAWRLPLPASMDTMDIRRAAA
EAAEGFRPAEFGGLSSGSSDEKEMNLSVDMEKNSSLCLFYLDEEEMFDMPRLIDNMAQGLLLSPPQCSAG
YLNWDDVETEADAKLWSFSI

>RcDREB1A gi|255537271|ref|XP_002509702.1| [Ricinus communis]
MDHHIFSQLSDPFPFNSNSITRDSPTLSDACSAPGGSSHSDEEVILASNLPKKPAGRRVFKETRHPVFRG
VRRRNGNKWVCEVRVPNKKSRIWLGTYPTPEMAARAHDVAALALRGKSACLNFSDSAWRLPIPSSTSARE
IRRIAAEAAESFRPQEFGCPGEQNSSTEDHPSGACCDESKTTVPFMDAEEVFDMPSLLVEMAQGLLLSPL
RFEEDVGTDWNGLENDFDISLWSD

>AtCBF4 gi|15242244|ref|NP_200012.1| [Arabidopsis thaliana]

MNPFYSTFPDSFLSISDHRSPVSDSSECSPKLASSCPKKRAGRKKFRETRHPIYRGVRQRNSGKWVCEVR
EPNKKSRIWLGTFPTVEMAARAHDVAALALRGRSACLNFADSAWRLRIPETTCPKEIQKAASEAAMAFQN
ETTTEGSKTAAEAEEAAGEGVREGERRAEEQNGGVFYMDDEALLGMPNFFENMAEGMLLPPPEVGWNHND
FDGVGDVSLWSFDE

>EgCBF1D gi|193161381|gb|ACF15447.1| [Eucalyptus gunnii]
MAAPGNFPDEEVRLASHHPKKRAGRKKFRETRHPVYRGVRLRDSGKWVCEVREPRKKSRIWLGTFPTVEM
AARAHDVAALALRGRSACLNFVDSAWRLPVPASADTKDIQKAAAKAAVAFQPVESESEDVMSGDEKKSPS
EEGMLFDDEDVFGMPGLLTNMAEGMLLPPPRCGGDGYGGEDDGNLDAYVSLWSYSL

>BpCBF2 gi|145952336|gb|ABP98988.1| [Betula pendula]
MDVFSQYSWESESGAMHLSDEEIQLASRNPKKRAGRTKFKETRHPVYRGVRRRNSGKWVCEVREPNKQSR
IWLGTFPTAEMAARAHDVAAIALRGNSACLNFADSAWRLPLPASGTAKDIQRTAVEAAEAFRPTETKAVE
ERQPSEGVFFMDEEAVFGMPGLLVNMAEGMLLPPPYCVGDDDVYGGDDVEAHADVSLWSYSI

>LhCBF3 gi|171191040|gb|ACB45078.1| [Solanum habrochaites]
MFYSDPRIESSSSDSFRANHSDEEVILASNNPKKPAGRKKFRETRHPVYRGVRKRNSGKWVCEVREPNKK
SRIWLGTFPTAEMAARAHDVAAIALRGRSACLNFADSAWRLPTPASSDTKDIQKAAAEAAESFRPLKSEE
EESVVKDQSTTPDDMFFMDEEALFCMPGLLTNMAEGLMVPPPQCTEMGDHVEADDMPLWSYSI

>VrCBF1B gi|39578540|gb|AAR28672.1| [Vitis riparia]
MDSDHEEFSASSSSSSSRTSSNPSDSLLPLQCIGHKRKAGRKKFRETRHPIYRGVRQRNGNKWVCEVREP
LKKSRIWLGTFPTPEMAARAHDVAALALRGRFASLNFSDSAWRLPRPKSSSAEDIQVAALEATKAFNPTA
PSSSSLASALDNMSGVADSKKVLETSPNVESPKLKSQRMVLEVSPVDTKRSEKVGDGSTPVFMDEEAMFN
MQGLINSMAEGLLLTPPAMCKGFSWDDATDSHIDLSLWNDD

>VaCBF1 gi|156186801|gb|ABU55657.1| [Vitis aestivalis]
HEEFSASSSSSSSRTDSNPSDSLLPLQCIGHKRKAGRKKFRETRHPIYRGVRQRNGNKWVCEVREPLKKS
RIWLGTFPTPEMAARAHDVAALALRGRFASLNFPDSAWRLPRPKSSSAEDIQVAALEATKAFNPTAPSSS
SLASALDNMSGVADSKKVLETSPNVESPKLKSQRMVLEVSPVDTKRSEKVGDGSTPVFMDEEAMFNMQGL

>VrCBF1A gi|39578538|gb|AAR28671.1| [Vitis riparia]
MDSDHEEFSASSSSSSSRTNSNPSDSLLPLQCIGHKRKAGRKKFRETRHPIYRGVRQRNGNKWVCEVREP
LKKSRIWLGTFPTPEMAARAHDVAALALRGRFASLNFPDSAWRLPRPKSSSAEDIQVAALEATKAFNPTA
PSSSSLASALDNMSGVADSKKVLETSPNVESPKLKSQRMVLEVSPVDTKRSEKVGDGSTPVFMDEEAMFN
MQGLINSMAEGLLLTPPAMCKGFSWDDATDSHIDLSLWNDD

>VvCBF1 gi|39578542|gb|AAR28673.1| [Vitis vinifera]
MDSDHEEFSASSSSSSSRTNPNSSDSLLPLQCIGHKRKAGRKKFRETRHPIYRGVRQRNGNKWVCEVREP
LKKSRIWLGTFPTPKMAARAHDVAALALRGRFASLNFPDSAWRLPRPKSSSAEDIQVAALEATKAFNPTA
PSSSSLASALDNMSGVADSKKVLETSPNVESPKLKSQRMVLEVSPVDTKRSEKVGDGSTTVSMDEEAMFN
MQGLINSMAEGLLLTPPAMCKGFSWDDATDSHIDLSLWNDD

>VvCBF3 gi|254952727|gb|ACT97164.1| [Vitis labrusca x Vitis vinifera]

MESERDQSSPSSSSSSSQTKCSISSSPVHKRKAGRKKFRETRHPVYRGVRQRNGNRWVCEVLDPKNKSRI
WLGTFPAPEMAARAHDVAALAFRGDFAALNFPDSASRLPRAKSSSARDIQVAALAAAMAFRPAAPSSSSS
HISHVTACSEELETSCSEDSPQLESRKKVVGVTLEDSESSQSAPHGSSTVFMDEEALFNMPGLINSMAEG
LLLAPPTMLGGFSWDDTTSYTDLSLWNDD

>RcCBF gi|255582828|ref|XP_002532187.1| [Ricinus communis]
MEKNDSSSTCKLSPCSPCSHISHQATLAPAKKRKAGRTKFKETRHPIYRGVRRRNGNKWVCEVREPNLKS
RIWLGTYPTPEMAARAHDVAALAFRGEFASLNYLDSAWILPRPKSSSHEDIKRAALEAAEAFKPSSTDLS
STSPPSSSSCSSASSKPSHEDDLRFSLMDTQDENEKKVLNNGNSSSMNIEPCPNVSIEIRNECSVTSFLD
DEALFNMPVLLDSMAEGLILTPPSIRRGFNWDDMAFAVDLTLWRD

>VrCBF2 gi|39578544|gb|AAR28674.1| [Vitis riparia]
MDLDRESSASSTSSPSRANLVSSDPRCPSRCIPHKRKTGRKKFRKTRHPIYRGVRQRNENKWVSEVREPS
KKSRIWLGTFPTPEMAARAHDVAALALRGHFASLNFPDSAWRLPRARSSSAGDVQFAAIQAAKAFQQPPS
SSSSTPFVMDNMSAGSRKILETSSVVDTPQLKSQKKVVGVSSMDSKSWEKAGDGFPTAFVDEEAVFNMPG
LIDSMAEGLLLTPPAMCEGFSWDDAVSHIDLSLWNHDFLS

>PtCBF6 gi|134038598|gb|AB048367.1| [Populus trichocarpa]
MCLLCPTYVTSCFMKNKAGRKKFKETRHPVYRGVRRRNGNKWVCEVREPNKKSRIWVGTFKSPEMAARAH
DVAALALKGELAALNFLDSALILPRAKSSSARDIQRAALAATEVLGRSASSCSSSSSPDHKKLSCVPNHS
NATFFDEEALFNMPGLLDSMAEGLILTPPAMARGVYWDDMACSTDLTLWEDDYLDSQ

>ShCBF2 gi|171191053|gb|ACB45088.1| [Solanum habrochaites]
MDIFESYYSNLLVESSLLSSLSMSDTNNINHYSPNEEIILASNYPKKPAGRKKFRETRHPVYRGVRKRNS
GKWVCEVREPNKKSRIWLGTFPTVEMAARAHDVAALALRGRSACLNFADSAWRLPIPASSNSKDIQKAAA
EAAEIFRPLKESEEVSGESPETSENVQESSYFVDEEALFFMPGLLANMAEGLMLPPPQCLEIGDHYVELA
DVHAYMPLWDYSI

>StCBF2B gi|171191066|gb|ACB45098.1| [Solanum tuberosum]
MDIYYSDPLAESSISDTNNNHYSPNNFSDEEVINLASNNPKKPAGRKKFRETRHPVYRGVRMRNSGKWVC
EVREPNKKSRIWLGTFPTVEMAARAHDVAALALRGRRSACLNFADSAWRLPIPASSNSKDIQKAAAEAAE
IFRPESEEVSGECSSSTTPETLENTFFMKEESSLFMDEEALFYMPGLIVNMAEGLMLPPPQCLEIGDHYV
ELVDMHAYMPLWNYSI

>StCBF4 gi|171191046|gb|ACB45083.1| [Solanum tuberosum]
MDQLNKNSSLILASNNPKKPAGRKKFRETRHPVYRGVRMRNSGKWVCEVREPNKKSRIWLGTFPTAEMAA
RAHDVAALALRGRSACLNFADSAWRLPIPASSNSKDIQKAAAEAAEIFRPSEESERVASSEMHESIFFMN
DEGRESSFFMDEEALFDMPGLIANMAEGLMLPPPQCAEVEDHYYMEADDAYMPLWNY

>LeCBF1 gi|18535580|gb|AAK57551.1| [Solanum lycopersicum]
MNIFETYYSDSLILTESSSSSSSSSFSEEEVILASNNPKKPAGRKKFRETRHPIYRGIRKRNSGKWVCEV
REPNKKTRIWLGTFPTAEMAARAHDVAALALRGRSACLNFSDSAWRLPIPASSNSKDIQKAAAQAVEIFR
SEEVSGESPETSENVQESSDFVDEEAIFFMPGLLANMAEGLMLPPPQCAEMGDHCVETDAYMITLWNYSI

>StCBF2A gi|171191065|gb|ACB45097.1| [Solanum tuberosum]

MFPSYYSEPLAELSPSSSSSSISDNSNHYYPNNNFSDEEVINLASNNPKKPAGRKKFRETRHPVFRGIRM
RNSGKWVCEVREPNKKSRIWLGTFPTAEMAARAHDVAALALRGRSACLNFADSVWRLPIPASSNSKDIQK
AAAEAAEIFRPSESEEVSGECSNITTPETPEKALSMNEEAQVNSFFMDEEALFYMPGLIANMAEGLMLPL
PQCLEIGDYVEADHAYISLWNYSI

>HbCBF1 gi|66269671|gb|AAY43213.1| [Hevea brasiliensis]
MDVFPQYSDSLPFATHSCSLHYPESSTLSDTCSALRANLSDEEVLLASSYPKKRAGRKKFRETRHPIYRG
VRRRNSGKWVCEIREPNKKSRIWLGTFPTEEMAARAHDVAALALRGRSACLNFADSSWRLPVPASREAKD
IRKAAAEAAMAFQPEGTEGFSGELKQENKWTTESAPEDVFYMDEEAVFAMPGLLASMAEGMLLPPPQCVA
GSGGEDGEMDAADVSLWSFSI

>StCBF3 gi|212525374|gb|ACJ26758.1| [Solanum tuberosum]
MDIFRSYYSDPQIESSSSFSDTTTYNSPNNRANHSDEEVILASNNPKKPAGRKKFRETRHPVYRGVRMRN
SGKWVCEVREPNKKTRIWLGTFPTAEMAARAHDVAAIALRGRSACLNFADSAWRLPTPASSDTKDIQKAA
AEAAESFRPLKSEEESVVTTGDQSSTPDDMFFMDEEALFCMPGLLTNMAEGLMVPPPQCTEMGDHVEADD
MPLWSYSI

>PtDREB68 gi|224058611|ref|XP_002299565.1| [Populus trichocarpa]
MPNDRQEASSFSDSSTSRRVVHSDEEVLLATSFPKKRAGRRIFRETRHPVFRGVRKRNGNKWVCEMREPN
KKSRIWLGTYPTPEMAARAHDVAALALRGKSACLNFADSAWRLPVPVSKDSKDITRAANEAAELFRPQEF
GGHPAKQQDSNAEAVFAMPGENDVFFEEAVFDMPGLLVDMAEGLLLSPPRYVRNDCNDLDHMENGSDLSL
WSY

>RhDREB1B gi|209171188|gb|ACI42860.1| [Rosa hybrid cultivar]
MDGGFSAFPQYYDHNTQYPFGSDLPESSSHSDNNSGNGVGRMNLSDEEVMLAATYPKKRAGRKKFKETRH
PVYRGVRRRNSGKWVCEVREPNKKTRIWLGTFPTAEMAARAHDVAAISLRGRSACLNFADSAWRLPVPAS
TSAKDIQTAAAQAAEAFRPTGEESEVVSGSVTATEAELEPVFCMDEEDVFGMPGLLANMAEGMLLSPPHY
NSYGGDDMEGYADVSLWSYSI

>StCBF5 gi|171191045|gb|ACB45082.1| [Solanum tuberosum]
MDQLNKNSSLILASNNPKKPAGRKKFRETRHPVYRGVRMRNSGKWVCEVREPNKKSRIWLGTYPTAEMAA
RAHDVAAIALRGGSACLNFADSAWRLPTPASSDTKDIQKAAAEAAESFRPLKSEEESVVSTGDQSSTPDN
MFFMDEEALFCMPGLLTNMAEGLMVPPPQCTEMGDHVEADDMPLWSYSI

>PtCBF4 gi|145322756|gb|ABP64695.1| [Populus trichocarpa]
MPNDRQEASSFSDSSTSRRVVHSDEEVLLATSFPKKRAGRRIFRETRHPVFRGVRKRNGNKWVCEMREPN
KKSRIWLGTYPTPEMAARAHDVAALALRGKSACLNFADSAWRLPVPVSKDSKDITRAANEAAELFRPQEF
GENDVFFKEAVFAMPGENDVFFEEAVFDMPGLLVDMAEGLLLSPPRYVRNDCNDLDHMENGSDLSLWSY

>NtDREB4 gi|190333406|gb|ACE73696.1| [Nicotiana tabacum]
MNIFGDHNFDPLIPTLSTSLLPAAESSTSSDSGSSGSTPNYSDEEVMLASNYPKKRAGRKKFRETRHPVY
RGIRRRNSNKWVCEVREPNKKSRIWLGTFPTAEMAARAHDVAAIALRGRSACLNFADSAWRLPIPTSAAA
KDIQKATVEAAETFRPVESHRENFKEIIVDQVIQELVAELPDNVLFMDEEALFCMPRLLVNMAEGLMLPP
PQCIIDGYEMEADHADMSLWSYINSMAEGLLLTPPAMCKGFSWDDATDSHIDLSLWNDD

>PtDREB69 gi|224053977|ref|XP_002298067.1| [Populus trichocarpa]
MVMGGSNSFSPDKQESSLSSLLSDSSGSQQDSPSSNEKVLLATSRPKKRAGRRIFKETRHPIFRGVRKRN
GDKWVCELREPNKKSRIWLGTYPTPEMAARAHDVAALAFRGKSACLNFADSAWRLPVPISNEAKDIRRAA
SEAAELFRTSDLGGQVMEDFRREDRGEVCSSTNDDIRDLPSENVGYIDEEAEFNMPGLLASMAEGLLLSP
PHYTGDWNDGEIDADWSLWSS

>BpCBF1 gi|145952334|gb|ABP98987.1| [Betula pendula]
MDVFSQYSSESESGAMHLSDEEIRLASRNPKKRAGRKKFKETRHPVYRGVRRRNSGKWVCEVREPNKQSR
IWLGTFPTAEMAARAHDVAAIALRGNSACLNFADSAWRLPVPASGTAKDIQRTAAEAAEEFRPAESKAVE
DRQPSESLFFMDEEAVFGMPGLMINMAEGMLLPPPYCVGDDDGYGGDVMEAHAEVSLWSYSI

>PtDREB67 gi|224131624|ref|XP_002328068.1| [Populus trichocarpa]
MEFENHSPSISSLRQNLSCKPCNNPINYIYNLKQAQERPVTVLKKKKAGRKKFKETRHPVYRGVRKRNGN
KWVCEVREPNKKSRIWLGTFTSPEMAARAHDVAALALKGETATLNFPDSALILPRAKSSSAGDIRRAARD
AALNDDPFSMVMMSCPNNCSEKVSHQPNALFFDEEALFNMPGLLDSMAEGLILTPPVMQRGFNWDDMACS
TDLTLWEED

>SlCBF1 gi|267799530|gb|ACY79412.1| [Solanum lycopersicoides]
MNIFETYYNSDSLILIESSSSSSSSSSSFSEEEIILASNNPKRPAGRKKFRETRHPIYRGVRMRNSGKW
VCEVREPNKKTRIWLGTFPTAEMAARAHDVAALALRGRSACLNFSDSAWRLPIPASSNSKDIQKAAAEAA
EIFRPSLSESEEVSGESDNSTTPETPENVNEEVQESSFFVDEEALFFMPGLLANMVEGLMLPPPQCAEMG
DHYVETDAYMITLWNYSI

>LhCBF1 gi|72068957|dbj|BAE17131.1| [Lycopersicon hirsutum]
MNIFETYNSDSLISTESSSSSSSSSSSLFSEEEIILASNNPKRPAGRKKFRETRHPIYRGIRKRNSGKWV
CEVREPNKKTRIWLGTFPTAEMAARAHDVAALALRGRSACLNFSDSAWRLPIPASSNSKDIQKAAAEAAE
IFRPLKESEEVSGESDNSTSPETSENVQESSDFVDEEAIFFMPGLLANMAEGLMLPPPQCAEMGDHYVET
DAYMITLWNYSI

>ShCBF1 gi|171191052|gb|ACB45087.1| [Solanum habrochaites]
MNIFETYNSDSLISTESSSSSSSSLFSEEEIILASNNPKRPAGRKKFRETRHPIYRGIRKRNSGKWVCEV
REPNKKTRIWLGTFPTAEMAARAHDVAALALRGRSACLNFSDSAWRLPIPASSNSKDIQKAAAEAAEIFR
PLKESEQVSGESDNSTSPETSENVQESSDFVDEEAIFFMPGLLANMAEGLMLPPPQCAEMGDHYVETDAY
MITLWNYSI

>SlCBF3 gi|45826358|gb|AAS77819.1| [Solanum lycopersicum]
MFYSDPRIESCSSFSDSIRANHSDEEVILASNNPKKPAGRKKFRETRHPVYRGVRKRNSGKWVCEVREPN
KKTRIWLGTFPTAEMAARAHDVAAIALRGRSACLNFADSAWRLPTPDSSDTKDIQKAAAQAAEIFRPLKS
EEEESVVKDQSTTPDDMFFMDEEALFCMPGLLTNMAEGLMVPPPQCTEMGDHVEADDMPLWSYSI

>PtDREB70 gi|224122476|ref|XP_002318846.1| [Populus trichocarpa]
MDLFSHYSDPSPFGATDFWSVFNENNGINQEQCSYSPVLSDSSISSNVTTRVQPAPNFSDEEVMLASRNP
KKRAGRKKFRETRHPVYRGVRRRNSGKWVCEVREPNKKSRIWLGTFPTAEMAARAHDVAALALRGRSACL
NFADSAWRLPVPASSEAKDIQKAAAEAAGGFRPEGCVGGELMRTGDEGEKAAETTAEAGEEVFYMDDEAV
FGMPGLLANMAEGMLLPPPHCGGGGDGWDNMENIDADMPLWSFSI

>PtCBF1 gi|134038590|gb|ABO48363.1| [Populus trichocarpa]
MLASRNPKKRAGRKKFRETRHPVYRGVRRRNSGKWVCEVREPNKKSRIWLGTFPTAEMAARAHDVAALAL
RGRSACLNFADSAWRLPVPASSEAKDIQKAAAEAAGGFRPEGCVGGELMRTGDEGEKAAETTAEAGEEVF
YMDDEAVFGMPGLLANMAEGMLLPPPHCGGGGDGWDNMENIDADMPLWSFSI

>AbCBF1 gi|156186811|gb|ABU55662.1| [Ampelopsis glandulosa var. brevipedunculata]
TNPNSSNSLLPLQCISHKRKAGRKKFRETRHPIYRGVRQRNGNKWVCEVREPLKKSRIWLGTFPTPEMAA
RANDVAALALRGHFASLNFPDSAWSLPRPKSSAPEDIQVAALEATKAFNPTAPSSSSLSSASDSMSGVAG
SKKVLETSLSVDSPNLLTQRKVLEVSSVDSKRSEKVGDGSMTVFMDEEALFNMPGLIDSMAEGLIL

>CaCBF2 gi|156186831|gb|ABU55671.1| [Cissus antarctica]
VNPVSSDSRWSSRCIPHKRKNGRKKFRETRHPIYRGVRQRNANKWVSEVREPRKKSRIWLGTFPTPEMAA
RAHDVGALSLRGHSTSLNFPDSAWLLPRASSSSVGDIQLAAIQAAKAFQQPASSSSSSSIVIDNMSTGSR
KILQTSSVVDSPHSKSQKKVSEASSVDSKRLEKAGDGFPTGFVDEEAVFNMPCLIDSMAEGLLL

>CaCBF1 gi|156186816|gb|ABU55664.1| [Cissus antarctica]
INPNSSDSLLLLQRIGPKRKAGRKKFRETRHPIYRGVRQRNGNKWVCEVREPFKKSRIWLGTFPTPEMAA
RAHDVAALALRGHFASLNFPDSAWRLPRPMSSSAEDIQVAALEATKAFNPTAPSSSSLSSALDNMSGVAD
SKKVLKTPPSVDSPKLKRQRKFLEVPSVDTKRSEKVGDGSITVFMDEEALFNMPGLIDSMAEGLLL

>PiCBF1 gi|156186807|gb|ABU55660.1| [Parthenocissus inserta]
LQCIGHKRKAGRRKFRETRHPIYRGVRQRNGNKWVCEVREPLKKSRIWLGTFPTPEMAARAHDVAALALR
GHFASLNFPDSAWRLPRPKSSSAEDIQIAALEATKSFNPTAPSSSSLASALDSMSGVPGSKKVLETSPSV
ESPKLKSQRKVLEVSSVDTKVADGSMTVFMDEEAVFNMPGLINS

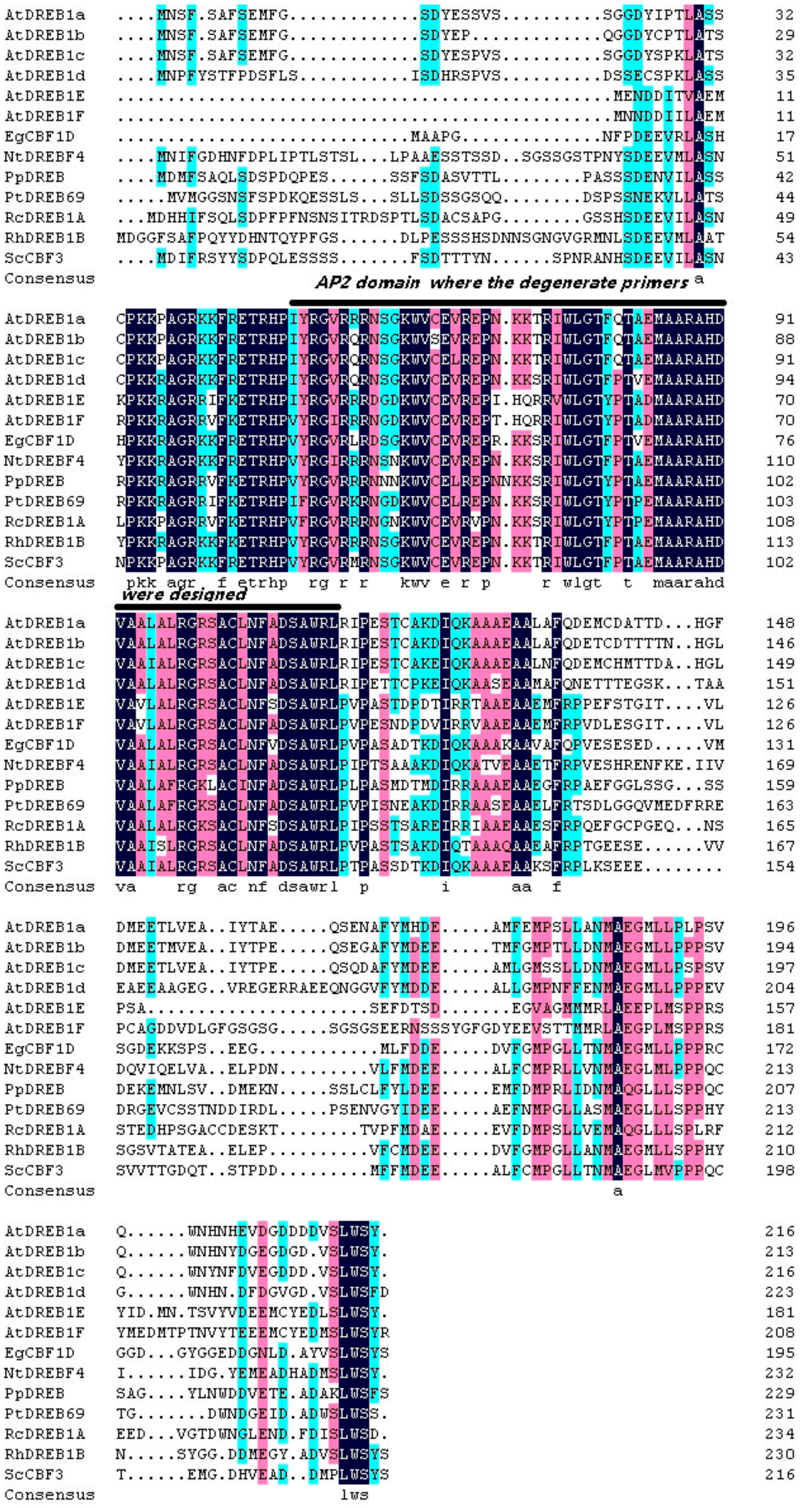

Figure S1. The conserved AP2 domain of some dicotyledon *DREB* genes.
